# Examination of Diagnostic Accuracy of UroVysion Fluorescence *In Situ* Hybridization for Bladder Cancer in a Single Community of Japanese Hospital Patients

**DOI:** 10.31557/APJCP.2019.20.4.1271

**Published:** 2019

**Authors:** Takashi Nagai, Takehiko Okamura, Takahiro Yanase, Ryosuke Chaya, Yoshinobu Moritoki, Daichi Kobayashi, Hidetoshi Akita, Takahiro Yasui

**Affiliations:** 1 *Department of Urology, Anjo Kosei Hospital, 28 Higashihirokute, Anjo-cho, Anjo, *; 2 *Department of Nephro-Urology, Nagoya City University Graduate School of Medical Sciences, Kawasumi 1, Mizuho-cho, Mizuho-ku, Nagoya, Japan. *

**Keywords:** Urinary bladder carcinoma, cystoscopy, FISH, cytology

## Abstract

**Objective:**

UroVysion (Abbott Molecular, Inc., Illinois, USA) is based on multicolor fluorescence in situ hybridization (FISH). It has been used successfully in the USA following its Food and Drug Administration approval in 2001. However, the technology was not approved for use in Japan until 2017. Cystoscopy and urine cytology are the most frequently used examinations to detect bladder cancer in Japan, and there are only a few reports regarding the performance of UroVysion. Therefore, the aim of this study is to examine the diagnostic accuracy of UroVysion FISH in Japanese patients whose tumors are detected by cystoscopy before transurethral resection of bladder tumor (TURBT).

**Methods:**

From April 2018 to July 2018, a total of 40 patients who were diagnosed as having bladder tumors by cystoscopy, and therefore underwent TURBT were registered in this study. One day before TURBT, urine cytology and UroVysion FISH were used in order to compare the accuracy with which they could detect bladder carcinoma, as confirmed by pathological results of TURBT.

**Results:**

The pathological results of TURBT showed urothelial carcinoma in 33 cases. Urine cytology showed positive results for 0 cases (0%), suspicious results for 10 cases (30.3%), and negative results for 23 cases (69.7%). On the other hand, UroVysion FISH indicated 9 positive cases (27.3%) and 24 negative cases (72.7%). There were 19 cases of urothelial carcinoma (57.6%) that were not detected by either method.

**Conclusion:**

We conclude that UroVysion FISH alone is insufficient to detect bladder cancer and that cystoscopy is essential for the optimum detection or follow up of bladder cancer cases in our hospital.

## Introduction

The global incidence of bladder cancer is 429,793 cases per year, and it is considered an international health problem (Mahdavifar et al., 2016), requiring an accurate diagnostic method. The standard diagnostic tools in Japan are cystoscopy and urine cytology. These tests are problematic, as cystoscopy is invasive, and urine cytology lacks sensitivity. The UroVysion test is based on multicolor fluorescence in situ hybridization (FISH) and is able to detect aneuploidy of chromosomes 3, 7, and 17, and the deletion of the 9p21 locus (Halling et al., 2000). It is reported that UroVysion FISH is superior in terms of sensitivity and is inferior in terms of specificity in a meta-analysis (Hajdinjak, 2008) and it has been used successfully in the USA following its FDA approval in 2001. As per the American Urological Association guidelines, UroVysion FISH has been approved for assessing responses to intravesical Bacillus Calmette-Guerin therapy and for determining the correct cytologies. As per the European Association of Urology guidelines, urinary biomarkers are recommended for use in detecting invisible tumors, particularly carcinoma in situ (CIS). In Asian countries, the indications for the use of UroVysion FISH have not been established. Moreover, there are few reports regarding the performance of UroVysion in Japan, since approval was just granted in 2017. Here, we examined the diagnostic accuracy of UroVysion FISH in patients whose tumors were detected by cystoscopy before transurethral resection of bladder tumor (TURBT).

## Materials and Methods

From April 2018 to July 2018, a total of 40 patients who were diagnosed as having bladder tumors by cystoscopy, and therefore underwent TURBT were registered in Anjo Kosei Hospital, Anjo, Japan. One day before TURBT, urine cytology and UroVysion (UroVysion Bladder Cancer Kit; Abbott Molecular, Inc., Des Plaines, Illinois) were used in order to compare the accuracy with which they could detect bladder tumors. Surgically resected materials were routinely fixed in 10% buffered formalin and embedded in paraffin for sectioning and histopathological assessment of hematoxylin and eosin-stained sections. Tumor grading and staging were performed with reference to the eighth edition of the Union for International Cancer Control tumor-nodes-metastases staging classification. Urine cytology was examined by one experienced pathologist and pathological results of TURBT were examined by one of two experienced pathologists in our hospital. Multivariate analyses were conducted with logistic regression analysis. A value of p<0.05 was considered as statistically significant. All the statistical analyses were performed using EZR.

The research was performed under approval of the institutional review board of Anjo Kosei Hospital (review board number: R18-060), and all patients provided informed consent.

## Results

The pathological results of TURBT showed urothelial carcinoma in 33 cases, including one case each of sarcomatoid variant and lymphoma-like variant carcinoma. There was one case of inverted papilloma, and no malignancy in the remaining 6 cases. Patient characteristics and tumor characteristics are shown in Table 1 and Table 2. 

The results of urine cytology and UroVysion are shown in Table 3. Urine cytology before TURBT showed positive results for 0 cases (0%), suspicious results for 10 cases (30.3%), and negative results for 23 cases (69.7%). On the other hand, UroVysion before TURBT indicated 9 positive cases (27.3%) and 24 negative cases (72.7%). There were 19 cases (57.6%) of urothelial carcinoma that were not detected by either method. The sensitivity of urine cytology and UroVysion are 30.3% and 27.3% respectively. The specificity of urine cytology and UroVysion are both 100%. The positive predictive value of both urine cytology and UroVysion are 100%, and the negative predictive value of urine cytology and UroVysion are 23.3% and 22.6%. 

The multivariate logistic regression analysis for UroVysion FISH positivity is shown in Table 4. The analysis revealed that high grade tumor tended to be an independent predictor of UroVysion FISH positivity (odds ratio [OR] = 6.18; 95% confidence interval [CI], 0.80-47.70, p=0.080). However, there were no significant differences with respect to other factors, such as high T stage (OR, 0.38; 95% CI, 0.030-4.79, p=0.45), recurrence (OR, 2.48; 95% CI, 0.31-19.70, p=0.39) and multifocal (OR, 0.757; 95% CI, 0.11-5.21, p=0.78).

## Discussion

The standard method for diagnosing bladder cancer in Japan is cystoscopy and urine cytology, even though cystoscopy is not ideal because it is invasive, and the sensitivity of urine cytology is not optimal. Accurate and non-invasive examinations are needed for diagnosing non-muscle invasive bladder cancer (NMIBC). We selected UroVysion FISH for evaluation, as it is one of the reliable urinary markers but there are few reports on its use in the Japanese population. 

**Table 1:** Patient Characteristics

Patient Characteristics		N
Male:Female, n (%)		24 (72.7):9 (27.3)
Median age (range)		77 (40-85)
History of upper tract urothelial carcinoma, n (%)		5 (15.2)
History of previous intravesical treatment, n (%)	BCG	3 (9.1)
	THP	1 (3.0)

BCG, Bacillus Calmette-Guérin; THP, pirarubicine hydrochloride

**Table 2:** Clinicopathologic Tumor Data

Clinicopathologic data		n (%)
Tumor stage	Tis	1 (3.0)
	Ta	27 (81.8)
	T1	4 (12.1)
	T2	1 (3.0)
Histologic stage	Low	14 (42.4)
	High	19 (57.6)
Onset	Initial	20 (60.6)
	Recurrent	13 (39.4)
Multifocality	Solitary	15 (45.5)
	Multifocal	18 (54.5)

**Table 3:** The Results of Urine Cytology and UroVysion

		Urothelial carcinoma	
		(+)	(-)
Urine cytology	Positive	0	0
	Suspicious	10	0
	Negative	23	7
UroVysion	Positive	9	0
	Negative	24	7

**Table 4:** The Results of Multivariate Analysis for UroVysion FISH Positivity

Parameter	OR	95%CI	P value
T stage (Tis, Ta vs T ≥ 1)	0.38	0.030-4.79	0.45
Grade (low vs high)	6.18	0.80-47.70	0.08
Onset (initial vs recurrent)	2.48	0.31-19.70	0.39
Multifocality (Solitary vs multifocal)	0.76	0.11-5.21	0.78

The literature regarding the effectiveness of UroVysion FISH is varied. Fritsche et al., (2010) described the diagnostic power of UroVysion FISH in cases of high-grade urothelial carcinoma and concluded that it is beneficial for patients with previous CIS, a high risk for the development of CIS, or previous unequivocal cytology suggestive of CIS, especially during or shortly after instillation therapy. Moreover, Ho et al., (2013) reported that UroVysion FISH has a high specificity (83.4%) for the patients with hematuria and it facilitates conservation of health resources and minimizes trauma by deferring cystoscopic or ureteroscopic examinations. Kojima et al., (2018) reported in their prospective, blinded, comparative study for the Japanese that UroVysion FISH had a higher sensitivity than urine cytology to detect bladder cancer after TURBT. On the other hand, there are some reports insisting that urine cytology and UroVysion FISH are comparable. Sullivan et al., (2009) concluded that both cytology and UroVysion have comparable specificity in cystoscopically negative cases. Lavery et al., (2017) also reported that there was no significant difference between sensitivity and specificity. Gomella et al., (2017) reported that UroVysion FISH showed a higher detection of urothelial carcinoma and worse specificity for bladder cancer compared to voided cytology. On the other hand, Krause et al., (2006) reported high sensitivity of UroVysion FISH, but they concluded that routine UroVysion use is not always recommended because of its cost. 

In our study, the number of patients with Tis was only 1 such that the clinical setting was unfavorable for detecting tumors by UroVysion FISH. Multivariate analysis showed that there were no significant relationships between UroVysion FISH positivity and the clinicopathological data. However, high grade tumors tended to be positive for UroVysion FISH, a result which was compatible with the previous reports. 

This study could not demonstrate the superiority of UroVysion FISH over urine cytology for the detection of bladder cancer in our hospital. Our study suggests that the detection rate of bladder cancer is equivalent when either urine cytology or UroVysion FISH is used. There are some limitations in this study. Firstly, the sample size of this study was small. Second, the subjects had a heterogenous background. These limitations might contribute to our study results indicating lower sensitivity than previous studies. 

In conclusion, combined urine cytology and UroVysion FISH detected almost 40% of urothelial carcinoma cases, but 60% of the cases were not detected. We conclude that UroVysion FISH alone is insufficient to detect bladder cancer, and that cystoscopy is essential for the optimum detection or follow up of bladder cancer cases in a real-world setting. The impact of the UroVysion FISH in clinical practice is limited and almost same result to urine cytology in this study. Combination of UroVysion FISH and urine cytology is considered a feasible method for the detection of NMIBC.

## Statement conflict of Interest

The authors declare no conflicts of interest.
